# A retrospective study on the usefulness of the JJ risk engine for predicting the incidence rate of coronary heart disease in type 2 diabetes patients

**DOI:** 10.1186/s13104-021-05508-9

**Published:** 2021-03-09

**Authors:** Yasunari Yamashita, Rina Kitajima, Kiyoshi Matsubara, Gaku Inoue, Hajime Matsubara

**Affiliations:** 1 Division of Clinical Pharmacy (Laboratory of Pharmacy Practice and Science III), and Research and Education Center for Clinical Pharmacy, School of Pharmacy, Kitasato University, 9-1, Shirokane 5-Chome, Minato-ku, Tokyo, 108-8641 Japan; 2 AdvanceSoft Corporation, 4-3, Kandasurugadai, Chiyoda-ku, Tokyo, 101-0062 Japan; 3 Department of Pharmacy, Kitasato University Kitasato Institute Hospital, 9-1, Shirokane 5-Chome, Minato-ku, Tokyo, 108-8641 Japan

**Keywords:** JJ risk engine, Type 2 diabetes, Coronary heart disease, Discrimination, Calibration, Hypoglycemia, C-statistic

## Abstract

**Objective:**

In 2018, we conducted a retrospective survey using the medical records of 484 patients with type 2 diabetes. The observed value of coronary heart disease (CHD) incidence after 5 years and the predicted value by the JJ risk engine as of 2013 were compared and verified using the discrimination and calibration values.

**Results:**

Among the total cases analyzed, the C-statistic was 0.588, and the calibration was p < 0.05; thus, the JJ risk engine could not correctly predict the risk of CHD. However, in the group expected to have a low frequency of hypoglycemia, the C-statistic was 0.646; the predictability of the JJ risk engine was relatively accurate. Therefore, it is difficult to accurately predict the complication rate of patients using the JJ risk engine based on the diabetes treatment policy after the Kumamoto Declaration 2013. The JJ risk engine has several input items (variables), and it is difficult to satisfy them all unless the environment is well-equipped with testing facilities, such as a university hospital. Therefore, it is necessary to create a new risk engine that requires fewer input items than the JJ risk engine and is applicable to several patients.

## Introduction

Currently, a risk engine that calculates the probability of CHD in type 2 diabetes patients is a useful prediction method. The Japan Diabetes Complications Study (JDCS)/the Japanese Elderly Diabetes Intervention Trial (J-EDIT) risk engine (JJ risk engine) was developed in Japan in 2012. This risk engine was developed using Japanese patient data; therefore, it is expected that its prediction accuracy will be higher for Japanese subjects than for Western subjects [1–3]. The JJ Risk Engine accurately predicts macro- and micro-vascular complications and provides useful information for risk classification and health economic simulations. The algorithm of this risk engine uses the multi-state Cox regression model [4].

However, since 2012 when the JJ risk engine was developed, diabetes treatment policies have changed significantly. Before 2010, a high premium was placed on decreasing blood sugar levels while treating diabetes, making it difficult to avoid hypoglycemia. Moreover, since 2010, hypoglycemia, particularly severe hypoglycemia, has become a recognized risk factor for the development of coronary heart disease (CHD) [5]. With the announcement of the “Kumamoto Declaration 2013,” the diabetes treatment policy has changed to one that emphasizes the prevention of hypoglycemia [6–8]. Accordingly, the use of antidiabetic drugs that are less likely to cause hypoglycemia has increased.

Therefore, given these changes regarding the diabetes treatment policy and the use of antidiabetic drugs, it can be considered that the frequency of hypoglycemia is lower currently than at the time when the JJ risk engine was developed. Hence, the number of CHD cases predicted by the JJ risk engine and the actual number may be different because the JJ risk engine predicted the risk of CHD using the data of patients receiving treatments that are less likely to cause hypoglycemia.

Here, we aimed to compare the JJ risk engine-predicted values with the actual complication incidences to verify whether changes in the diabetes treatment policy affected the incidence of complications in type 2 diabetes patients.

## Main text

### Methods

#### Patients

*Selection criteria*: The subjects were patients with type 2 diabetes who visited the Kitasato University Kitasato Institute Hospital, Diabetes Center, from January 2013 to December 2013, and were on continuous treatment for the following 5 years, until 2018.

*Exclusion criteria*: Patients who refused to participate in the study or had any of the following histories were excluded: angina, myocardial infarction, stroke, peripheral arterial disease, familial hypercholesterolemia, familial type III hyperlipidemia, nephrotic syndrome, renal diseases other than diabetic nephropathy, microhematuria, pre-proliferative and proliferative retinopathy, and major ocular diseases (e.g., glaucoma, dense cataract, or history of cataract surgery).

Further, patients with test values of serum creatinine > 1.3 mg/dL and urinary albumin ≥ 150 mg/gCr were also excluded.

Other exclusion criteria included patients who lacked the input items of the JJ risk engine and patients with non-type 2 diabetes such as borderline diabetes, type 1 diabetes, and gestational diabetes.

This study was conducted in accordance with the Ethical Guidelines for Medical and Health Research Involving Human Subjects. The Kitasato University Kitasato Institute Hospital, Research Ethics Committee, approved the study (Control number: 18061) and provided permission to review patient records as well as the use of the corresponding data. The option to opt-out of the study was provided to the patients at the start of the study (2018).

#### Statistical analysis

The incidence of CHD for each patient was calculated by entering patient data into the JJ Risk Engine web application [1].

Some assessment indices of the JJ Risk Engine are explained below:

Discrimination: an index that evaluates how accurately the presence or absence of an event can be predicted by a prediction model; the C-statistic, calculated on the basis of the receiver operating characteristic (ROC) curve, is used as a criterion for measuring the predictive accuracy [9, 10].

Calibration: an index to measure the degree of agreement between the prediction by the model and the actual outcome; the significance probability calculated by the Hosmer–Lemeshow test is used as the criterion for predictability. The significance level was set at 0.05 (p < 0.05) [10].

Sensitivity: Proportion of patients with the target condition who have a positive test result.

Specificity: Proportion of patients without the target condition who have a negative test result..

R version 2.5.1 (http://www.r-project.org, library Design, Hmisc, ROCR) was used for determining discrimination, calibration, sensitivity, and specificity, whereas the ROC curve and Hosmer–Lemeshow test were used for calculation [10, 11].

#### Comparative analysis

Observed value of CHD and predicted value by the JJ risk engine: all patients (verification 1):

To measure the degree of agreement between the observed value, which is the number of individuals developing CHD after 5 years (in 2018), and the predicted value by the JJ risk engine (as of 2013), the observed value/predicted value (O/P) ratio and the discrimination value were validated using calibration.

Observed value of CHD and predicted value by the JJ risk engine: frequency of hypoglycemia (verification 2):

Focusing on the risk of hypoglycemia, patients were categorized into two groups:

Group ①: Patients who use antidiabetic drugs prone to cause hypoglycemia alone (glimepiride, gliclazide, insulin).

Group ②: Patients not applicable to Group ①

The predictive ability of the JJ risk engine for CHD onset in each group was verified in the same way as verification 1, and comparisons were made between the two groups.

### Results

Patient baseline demographic and clinical characteristics are shown in Table 1.

**Table 1:** Baseline characteristics

Characteristics	All patients (n = 484)	Male (n = 329)	Female (n = 155)
Age (years)	64 ± 11	63 ± 10	65 ± 11
Height (cm)	164.2 ± 8.6	168.5 ± 6.2	155.3 ± 5.7
Weight (kg)	67.1 ± 13.7	71.2 ± 13.0	58.3 ± 10.5
Simple retinopathy; n (confirmed/total)	30/484	24/329	6/155
Atrial fibrillation; n (confirmed/total)	7/484	5/329	2/155
Presence of exercise habits; n (confirmed/total)	67/484	49/329	18/155
Current smoker; n (confirmed/total)	71/484	57/329	14/155
Disease duration (years)	11.8 ± 8.3	12.4 ± 8.4	10.6 ± 7.8
HbA1c (NGSP,%)	7.2 ± 3.0	7.3 ± 3.6	7.0 ± 0.8
Systolic blood pressure (mmHg)	125.2 ± 13.3	125.3 ± 13.3	124.9 ± 13.3
Total cholesterol (mg/dL)	188.8 ± 30.4	184.4 ± 30.0	198.1 ± 29.2
HDL cholesterol (mg/dL)	63.4 ± 17.4	60.5 ± 16.6	69.5 ± 17.4
Urinary albumin (mg/gCr)	22.2 ± 27.8	23.3 ± 29.7	20.0 ± 23.1

Data are expressed as means ± standard deviation

As shown in Table 2, the observed value was 15 individuals, and the predicted value by the JJ risk engine was 6.99 individuals; thus, the observed value was > the predicted value. The O/P ratio was 2.14, the C-statistic was 0.588, and the calibration was p < 0.05.

**Table 2:** Observed value of CHD and predicted value by the JJ risk engine: all patients

	n	Observed (individuals) (%; 95% CI)	Predicted (individuals)	O/P ratio	Discrimination; C-statistic (95% CI)	Calibration	Sensitivity	Specificity
All patients	484	15 (3.1; 1.7–5.1)	6.99	2.14	0.588(0.453–0.724)	p < 0.05	0.733	0.495

Similarly, in group ① (Table 3),the observed value was > the predicted value. On the other hand, in group ②, the O/P ratio was 0.81; therefore, the measured value ≒ the predicted value (Table 3).

**Table 3:** Observed value of CHD and predicted value by the JJ risk engine: frequency of hypoglycemia

	n	Observed (individuals)(%; 95% CI)	Predicted(individuals)	O/P ratio	Discrimination; C-statistic (95% CI)	Calibration	Sensitivity	Specificity
Group ①	282	13(4.6;2.5–7.8)	4.57	3.28	0.540(0.374–0.706)	–	0.385	0.799
Group ②	202	2(0;0–2.5)	2.46	0.81	0.646(0.565–0.728)	–	1	0.595

### Discussion

No large large-scale studies have been published showing fewer hypoglycemia cases after 2013 compared to the period before the development of the JJ Risk Engine. However, there is a growing number of drugs, such as sodium-glucose cotransporter 2 (SGLT2) inhibitors [12] and long-acting insulin [13], that are less likely to cause hypoglycemia when used alone, and the primary treatment strategy is to prevent hypoglycemia [6, 7].

These factors suggest fewer hypoglycemia cases today than before the development of the JJ risk engine.

Our study shows that the JJ risk engine could not correctly predict the risk of developing CHD, as indicated from the O/P ratios, discrimination, and calibration values. The calibration value could be calculated only in the “all patients” group, but not for the specifically divided patient groups because of the small sample size of the observed value therein [14].

It is assumed that the JJ risk engine predicts the risk of CHD onset in type 2 diabetes patients by considering the related risk factors and influencing factors. However, in our study, its risk predictive ability for the “all patients” group was poor; therefore, the effects of these factors may not be fully reflected in the prediction. Moreover, apart from the input items of the JJ risk engine, there are several risk factors and influencing factors involved in the development of CHD, such as high low-density lipoprotein (LDL) cholesterolemia, hypertriglyceridemia, family history of dyslipidemia, and hypoglycemia. The input items of the JJ risk engine do not include items related to hypoglycemia. Therefore, it was necessary to verify whether hypoglycemia affects the ability of the JJ risk engine to predict the onset of CHD.

As shown in Table 3, in group ① comprising patients expected to have a high frequency of hypoglycemia, the JJ risk engine could not correctly predict the risk of developing CHD, as evident from the O/P ratio, the discrimination, and the calibration values. In contrast, in group ② comprising patients expected to have a low frequency of hypoglycemia, the risk of developing CHD as predicted by the JJ engine is considered relatively accurate. In the current treatment policy, the priority is to prevent hypoglycemia. According to the Japan Diabetes Clinical Data Management Study Group's 2019 basic data [15], since 2013, HbA1c levels have stopped declining and have been on a gradual upward trend. It has been suggested that the concern regarding the prevention of hypoglycemia has led to poor control of HbA1c levels, which in turn has increased CHD risk. Therefore, we consider the observed value to be larger than the predicted value.

However, the actual state of hypoglycemia in each patient is not sufficiently reflected from these results. By classifying patients according to the risk of hypoglycemia in association with the use of antidiabetic drugs, we could not find a relationship between the predictive ability of the JJ risk engine for CHD onset and the frequency of hypoglycemia. We can specifically explain this with the results shown in Table 3; the JJ risk engine could not correctly predict the risk of developing CHD in group ① patients who were on SU drugs and low-dose insulin. However, the JJ risk engine predictability was high when tested in patients using high-dose insulin. To get closer to the actual state of hypoglycemia, it is necessary to further investigate hypoglycemia associated with the use of antidiabetic drugs, as we did in this study. We had further segregated group ① patients expected to have a high frequency of hypoglycemia based on their use of antidiabetic drugs. However, we were not able to verify this in the present study. In the SU drug group, there were 189 glimepiride users, of which more than 90% were using low doses (2 mg or less per day) [16], and there were 7 gliclazide users. Therefore, it was impossible to classify the SU drug group into a low-dose and a high-dose group. The insulin user group consisted of 80 patients, and the sample size was too small to analyze.

It was neither possible to find a relationship between the inaccuracy in the predictability of the JJ risk engine for CHD risk and the frequency of hypoglycemia nor identify the cause of the inaccuracy, and it is difficult to accurately predict the complication rate of patients using the JJ risk engine based on the diabetes treatment policy after the Kumamoto Declaration 2013.

The JJ risk engine has several input items, and it is difficult to fulfill them all unless the environment is well-equipped with testing facilities such as university hospitals. In particular, in Japan, urine albumin levels are rarely tested, which cannot be calculated unless a diagnosis of diabetic nephropathy has been made for insurance purposes [17, 18]. Therefore, it is necessary to create a new risk engine that requires fewer input items and is easily accessible to many patients. Since there are factors involved in CHD development other than those entered in the JJ risk engine, it would be useful to examine other factors as well [1].

### Conclusion

Our study shows that the JJ risk engine could not accurately predict the risk for CHD onset in patients with type 2 diabetes in our hospital. Moreover, it is necessary to create a new risk engine that can accurately predict the risk of developing CHD—with fewer input items and a simpler technique than the JJ risk engine—and aligns with the current diabetes treatment approach.

## Limitations

The relatively low number of people who develop CHD may affect the reliability of the analysis [19].

## Data Availability

The datasets generated and/or analyzed during the current study are not publicly available due to the privacy of the research participants but are available from the corresponding author on reasonable request. However, in this case, permission from The Kitasato University Kitasato Institute Hospital Research Ethics Committee is required.

## Abbreviations

CHD: Coronary heart disease
ROC: Receiver operating characteristic
HbA1c: Glycated hemoglobin
NGSP: National Glycohemoglobin Standardization Program
SU: Sulfonylurea
LDL: Low-density lipoprotein
